# Reduced Performance of Prey Targeting in Pit Vipers with Contralaterally Occluded Infrared and Visual Senses

**DOI:** 10.1371/journal.pone.0034989

**Published:** 2012-05-14

**Authors:** Qin Chen, Huanhuan Deng, Steven E. Brauth, Li Ding, Yezhong Tang

**Affiliations:** 1 Department of Herpetology, Chengdu Institute of Biology, Chinese Academy of Sciences, Chengdu, Sichuan, China; 2 Graduate University of Chinese Academy of Sciences, Beijing, China; 3 Department of Psychology, University of Maryland, College Park, Maryland, United States of America; 4 College of Life Sciences, Chongqing Normal University, Chongqing, China; University of Lethbridge, Canada

## Abstract

Both visual and infrared (IR) senses are utilized in prey targeting by pit vipers. Visual and IR inputs project to the contralateral optic tectum where they activate both multimodal and bimodal neurons. A series of ocular and pit organ occlusion experiments using the short-tailed pit viper (*Gloydius brevicaudus*) were conducted to investigate the role of visual and IR information during prey targeting. Compared with unoccluded controls, snakes with either both eyes or pit organs occluded performed more poorly in hunting prey although such subjects still captured prey on 75% of trials. Subjects with one eye and one pit occluded on the same side of the face performed as well as those with bilateral occlusion although these subjects showed a significant targeting angle bias toward the unoccluded side. Performance was significantly poorer when only a single eye or pit was available. Interestingly, when one eye and one pit organ were occluded on opposite sides of the face, performance was poorest, the snakes striking prey on no more than half the trials. These results indicate that, visual and infrared information are both effective in prey targeting in this species, although interference between the two modalities occurs if visual and IR information is restricted to opposite sides of the brain.

## Introduction

The pit organs of crotaline and boid snakes are unique sensory structures that can “see” targets through infrared (IR) receptors. As a result, the facial pit of pit vipers (Crotalinae) enables these snakes to accurately target endothermic prey animals even in absolute darkness [1], [2], [3]. Although some other species have been shown to detect IR radiation [3], the snake IR sensory system is the only known biological structure to form images using IR information [4], [5], [6]. Previous studies indicate that the infrared sense normally works in concert with other sensory systems to facilitate detection, localization, and capture of prey [3], [7], [8].

In pit vipers, blocking both the visual and IR sensory organs simultaneously disrupts predatory behavior, consistent with the idea that visual and/or infrared cues are necessary for optimal prey targeting [9]. Other studies show that the infrared organ (i.e. the facial pits of pit vipers and the labial pits of some pythons and boas) are as important as the eyes for orientation towards prey before strike initiation [10], [11]. Nevertheless the precise way these sensory systems are used during hunting is still unclear. Neuroanatomical studies have shown that visual and IR information are both projected to the contralateral midbrain tectum, converging on bimodal neurons in IR-sensitive snakes [12], [13], [14] thus raising the question of whether these sensory inputs work in a complementary way [15].

In the present study the effects of unilateral and bilateral ocular and/or pit organ occlusion on prey targeting were investigated in the short-tailed pit viper, *Gloydius brevicaudus*. The goals were to determine the importance of each sensory modality in prey targeting and capture and to determine if these functioned independently during hunting. The species was selected for study because of its hunting strategy. Field investigations and observations in the laboratory environment indicate that short-tailed pit vipers typically hold a fixed position while waiting to ambush prey thus making it relatively easy to measure head orientation and strike ranges. It is important to note that in their natural environment prey appear in the context of many different external conditions (such as light, temperature, and surroundings). Thus we hypothesized that the performance of prey capture will be less efficient following complete or partial visual and/or IR sensory deprivation.

## Results

**Figure 1:**
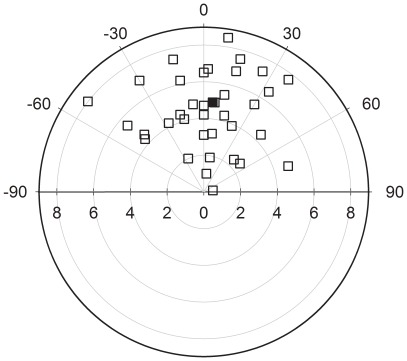
Performances of control conditions. Plots of all strikes (unfilled squares) for snakes in the control condition indicating strike distances (cm) and angles (°). The filled square represents the mean strike distance and angle in the control condition.

**Figure 2:**
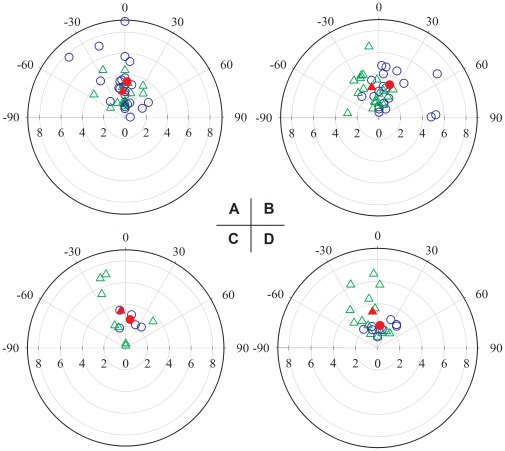
Performances of eight occluded conditions. Plots showing distances (cm) and angles (°) of individual strikes (unfilled symbols) and mean values (filled symbols) in the occlusion conditions: A: Binocular occlusion (blue unfilled circles and red filled circle) vs. bilateral pit organ occlusion (green unfilled triangles and red filled triangle). B: Left eye and pit occlusion (blue unfilled circles and red filled circle) vs. right eye-pit occlusion (green unfilled triangles and red solid triangle). C: Contralateral occlusion of the left eye and right pit organ (blue unfilled circles and red filled circle) vs. contralateral occlusion of the right eye and left pit organ (green unfilled triangles and red solid triangle). D: Unilateral opening of only the right pit (blue unfilled circles and red filled circle) vs. unilateral opening of only the left pit organ (green unfilled triangles and red filled triangle).

Parameter values are tabulated in Table 1. The distribution of strikes including strike distances, and angles are depicted in figures 1 and 2. All snakes used in the experiments exhibited strong motivation to hunt prey as indicated by repeated tongue flicks. Overall more than 95% of strikes were successful. On most trials, snakes only struck at prey once and waited for the prey to die before consuming it.

**Table 1:** Summary of parameters of prey attack for snakes in different occlusive conditions.

(Left/Right) Eyes	OO	XX	OO	XO	OX	XO	OX	XX	XX
(Left/Right) Pits	OO	OO	XX	XO	OX	OX	XO	OX	XO
Trials with strikes	20	15	15	15	14	6	7	13	9
Strikes number	36	23	21	23	19	6	8	14	11
Successful strikes	36	23	18	23	18	5	8	14	9
Strikes/trial	1.8	1.53	1.4	1.53	1.36	1	1.14	1.07	1.22
Latency time(mean ± sd)	94.8±68.5	239.2±282.0	157.7±208.2	130.3±96.7	204.9±197.1	149.7±121.5	183.1±229.1	159.3±188.4	134.8±58.1
Strike distance(mean ± sd)	4.9±2.0	3.3±2.3	2.4±1.2	3.2±2.2	2.9±1.4	2.6±0.64	3.5±2.7	3.3±1.9	2.0±0.6
Strike angle(mean ± sd)	6±32	4±31	−5±24	18±30	−13±26	9±22	−7±24	−8±24	6±26

*Note*: All of the data were obtained from video recordings. Strike angles (°) and Strike distances (cm) were measured from the last video frames right before the strikes occurred. The nine conditions are signified by combinations of “O” (open) and “X” (closed).

### Control Trials

All snakes performed perfectly capturing prey on each trial of the control condition. At least one strike occurred during each 20-minute trial period, yielding a total of 36 strikes (see Table 1). On nine control trials, snakes launched one strike. Snakes struck prey twice in eight trials. On one trial the snake struck three times and on two trials the snakes launched four strikes. An examination of the mice after these strikes revealed that for all trials in the control condition the snake killed the prey. These strikes were scored as successful strikes after reviewing the video recordings. Strike distances for control trials ranged from 0.5 to 8.5 cm (4.9±2.0 cm, Table 1). Strike angle varied over a broad range from the left to the right side of the snout (–52°to+81°; Fig. 1). Mean strike angle, however, did not show a significant bias toward either the left or right side (5.64°±31.95°, Table 1; Fig. 1, *P* = 0.359).

### Binocular Occlusion and Bilateral Pit Occlusion Trials

In order to investigate the efficiency of vision and IR in prey targeting, binocularly occluded trials and bilateral pit-occluded trials were conducted, and the results compared to controls and with each other.

#### Binocular occlusion trials

Binocularly occluded pit vipers struck prey in 15 of 20 trials. In all 15 of these strike trials the prey was bitten. In this condition snakes struck prey once in nine trials, twice in four trials, and three times in two trials. Thus there were 23 successful strikes in 15 trials in this condition (Table 1). The success rate for this condition was therefore 100% (see Table 1). Compared to the 20 control trials, the numbers of strikes and successful strikes were significantly lower than those in the control condition (*P* = 0.046; *P* = 0.046).Strike distance in the binocularly occluded condition ranged from 0.5 to 9 cm (3.3±2.3 cm, Table 1, Fig. 2A). Mean strike distance in this condition was not significantly less than that of control trials (*P* = 0.061).In the binocularly occluded group, strike angle varied, ranging from −43° to 90° (Fig. 2A). However, the mean strike angle in visually occluded trials was not significantly different from 0° (*P* = 0.9; Fig. 2A), and was not significantly different from that of control trials (*P* = 0.429; Fig. 2A). Therefore, strike accuracy and distance were not significantly different than controls, but fewer strikes were launched by snakes with both eyes occluded.

### Bilateral Pit Organ Occlusion Trials

In the bilateral pit organ occlusion condition, snakes struck at prey in 15 of 20 trials, the same as that of the bilateral ocular occlusion condition (Table 1). The success rate for this condition was 85.7% (see Table 1). The mean number of strikes per trial was significantly lower than that for the control condition (*P* = 0.025) and, the number of successful strikes was also significantly lower than for the control condition (*P* = 0.004). Strike distance (2.4±1.2 cm, Table 1) was substantially less than that in the control group (*P*<0.001; Fig. 2A). The strike angles ranged from –54° to 38°, including seven out of 18 strikes which were directed along the midline axis of the head (0° strike angle) (Fig. 2A). The mean strike angle in this condition was not significantly different from 0° (*P* = 0.465; Fig. 2A), and was not significantly different from that in the control trials (*P* = 0.225).

### Binocular Occlusion Trials vs. Bilateral Pit Organ Occlusion Trials

Strike trials, number of strikes and successful strikes were not significantly different between the two groups (*P*>0.05 in each case). Strike distance and strike angle in the binocularly occluded and bilateral pit organ occluded trials were not significantly different based on the results of nonparametric tests (*P* = 0.19 & *P* = 0.782). However, there was more variability in strike distance and angle for the binocularly occluded condition; snakes with bilateral pit organ occlusion struck at prey situated closer to the snout of the snake than those in the binocular occlusion condition (Fig. 2A).

### Behavior of Ipsilateral Visual/IR Sensory Deprived Snakes

The snakes with left eye and left pit occluded launched 23 strikes in 15 trials, while snakes with right eye and right pit occluded launched 19 strikes (with one miss) in 14 trials (Table 1). Both the number of strikes (L: *P* = 0.02; R: *P* = 0.021) and successful strikes (L: *P* = 0.02; R: *P* = 0.009) for both ipsilateral occlusion conditions were significantly lower than for controls. However, these parameters did not differ significantly between the left and right side occlusion conditions (*P* = 0.635; *P* = 0.455).

For the left side occlusion trials, strike distance (3.2±2.2 cm, Table 1) was significantly less than that of the control group (*P* = 0.027). The targeting angle was biased significantly toward the right side (18°±30°, *P* = 0.009; Table 1, Fig. 2B). In the right ocular/pit occlusion condition, strike distance (2.9±1.4 cm, Table 1) was significantly less than that of the control group (*P*<0.001). Targeting angle was biased significantly toward the left side (−13°±26°, Table 1; *P* = 0.049; Fig. 2B). Not surprisingly, the targeting angle was significantly different between the left and right side occlusion conditions (*P* = 0.003; Fig. 2B). On the other hand, targeting distance was not significantly different (*P* = 0.966; Fig. 2B) between the left- and right-sided visual/IR-deprived trial groups.

### Contralateral Ocular and Pit Organ Occlusion Conditions

In these conditions snakes attacked prey with the lowest efficiency (Table 1). In the left eye and right pit occluded condition, snakes struck once in six trials (with one miss) out of 20; in the right eye and left pit occluded condition, snakes struck prey on seven trials out of 20 (Table 1). The numbers of strikes and successful strikes were substantially lower than those in the control condition (*P*<0.001 for both comparisons). On the other hand, there were no significant differences in the numbers of strikes and successful strikes between the two contralateral occlusion conditions (*P* = 0.547; *P* = 0.426).

For the left eye and right pit occlusion trials, strike distance (2.6±0.6 cm, Table 1) was not significantly less than that for the control group (*P* = 0.188). Three strikes were launched to the open eye side (right, positive angle) and two strikes were launched to the open pit side (left, negative angle). Targeting angle was slightly biased toward the open eye side but this was not statistically significant (9°±22°, *P* = 0.438; Table 1, Fig. 2C).

For the right eye and left pit occluded trials, strike distance (3.5±2.7 cm, Table 1) was not significantly different from that of the control group (*P* = 0.547). Except for one strike directed towards the open pit side (right, positive angle), the other seven strikes were directed either straight ahead or to the open eye side (left, negative angle). Targeting angle was biased slightly towards the open eye side, but this was not significant (−7°±24°, *P* = 0.438; Table 1, Fig. 2C). There were no significant differences in targeting distance or angle between these two contralateral occlusion groups.

### Binocular Occlusion and Unilateral Pit Organ Occlusion

The snakes with only one pit open were able to attack prey successfully. Snakes with only the left pit open launched 14 strikes in 13 experimental trials, while those with only the right pit open struck 11 times (with two misses) in nine trials (Table 1). Numbers of strikes (Left: *P*<0.001; Right: *P*<0.001) and successful strikes (Left: *P*<0.001; Right: *P*<0.001) were significantly lower than for the control condition. Nonetheless, the numbers of strikes and successful strikes on 40 trials in the condition in which only one pit was open were significantly higher than for those in the 40 contralateral ocular/pit organ occlusion conditions (*P* = 0.022; *P* = 0.018). No significant differences for these two parameters were found between the two contralateral occlusion conditions (*P* = 0.465; *P* = 0.164).

For the left pit open trials, strike distance (3.3±1.9 cm, Table 1) was significantly less than that of the control group (*P* = 0.049). Targeting angle was biased slightly toward the open pit side (−8°±24°, *P* = 0.216; Table 1, Fig. 2D). For the right pit open trials, strike distance (2.0±0.6 cm, Table 1) was significantly less than that of the control group (*P* = 0.027). Targeting angle was slightly biased toward the open pit side (6°±26°, *P* = 0.547; Table 1, Fig. 2C). No significant differences in strike distance or strike angle were found between the two groups in which only one pit was open (*P* = 0.078; *P* = 0.426).

## Discussion

Prey catching in snakes is normally comprised of four steps: detecting, approaching, targeting and striking. Visual and IR sensitive systems are known to be involved in detecting and targeting prey in varied environments in these species [8], [16], [17]. For the short-tailed pit viper, hunting behavior seldom includes approaching prey presumably because this strategy minimizes energy consumption. For this reason short-tailed pit vipers usually wait unobtrusively for prey to come into strike range. Consequently a strike miss may incur substantial cost in the form of a lost meal. The ability to precisely target prey in *Gloydius* is therefore critical for survival. It is notable, therefore, that in the present study strike accuracy across both control and occlusion conditions was greater than 95% (Table 1) and that the main effect of sensory occlusion was a decrease in strike initiation. This is consistent with the idea that the snakes prefer to hold and adjust head position striking only at prey when targeting can be made precisely.

The present study shows that accurate prey targeting as reflected by rates of initiating successful strikes is significantly reduced by sensory deprivation. Occlusion of either eyes or pits or occlusion of one eye and one pit organ on the same side of the head resulted in about a 25% decrease in the initiation of successful strikes. Snakes in which only a single pit organ was available for hunting prey were able to initiate strikes on slightly more than half the trials. The poorest performance occurred in the contralateral occlusion condition, when one eye and one pit were occluded on opposite sides of the head and snakes did not launch successful strikes during more than half of the experimental trials.

The data are consistent with the idea that each sensory system (i.e. visual and IR) can compensate to a great degree if the other system is not available (see Table 1). This compensatory capacity would seem to be a valuable adaptation for a species which can hunt both during the day and at night. Thus pit vipers hunting during the day can make use of visual information although infrared imaging would be attenuated in daylight conditions with relatively high temperature. In contrast, pit vipers hunting in dark environments (during the night, or in caves), can make only limited use of vision while infrared imaging is clearest with lower background heat radiation. Pertinent to this we have observed that *Gloydius* snakes with both pit organs occluded do not launch strikes in a completely dark room (unpublished data). This is also consistent with the idea that in their natural environments both visual and IR systems act synergistically [16]. Interestingly, congenitally blind IR-imaging snakes aim and strike prey accurately under a variety of conditions suggesting the IR sense is sufficient for prey targeting in these species [18], [19].

In the ipsilateral eye and pit occlusion conditions, the snakes trended to launch strikes to the unoccluded side. Nevertheless successful strike initiation rates were not different from the binocular or bilateral pit occlusion conditions. Taken together these results support the idea that each system is nearly equally useful for prey targeting and capture.

It is striking that the poorest performance of the snakes in the present study occurred in the contralateral occlusion condition (see Table 1). Such a condition, in which visual and IR sensory input would be impaired on *opposite* sides of the head, would seem to be extremely unlikely to occur in the pit viper’s natural environment. However it is possible that information from the two sensory systems may receive conflicting information if prey are present in both the left and right sensory fields and moving in opposite directions or if prey moves rapidly across the field of convergence of the two eyes and pits. In order to avoid missing prey an inhibitory mechanism blocking strike initiation in these kinds of situations may hence be adaptive because unsuccessful strikes would be expected to result in escape of the prey.

**Figure 3:**
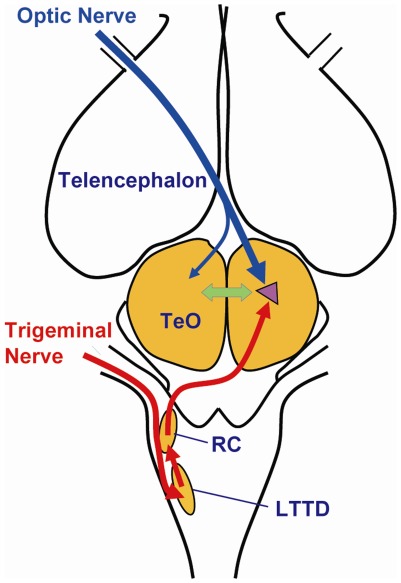
Schematic diagram of the two imaging sensory circuits. Schematic diagram of the snake brain (anterior is up and posterior is down) illustrating visual (blue) and IR (red) pathways showing convergence of visual (blue) and IR (red) inputs in the contralateral tectum. Abbreviations: LTTD, nucleus of the lateral descending trigeminal tract; RC, nucleus reticularis caloris; TeO, optic tectum. The arrow interconnecting the left and right optic tectums represents the tectal commissure and the filled triangle in the tecta represents the tectal bimodal neuronal population (see text).

Neuroanatomical and neurophysiological studies have shown that both visual and IR inputs converge on bimodal neurons in the optic tectum of pit vipers [10], [14], [15], [16] as summarized in Figure 3. These studies show that inputs derived from both sensory modalities form similar spatiotopic maps supporting the idea that direct interactions between them occur at this level of the snake brain [13], [15] consistent with the idea that the two sensory systems can compensate for one another or act synergistically [16]. Convergence of these tectal pathways may explain our observations that prey targeting accuracy for the ipsilateral occluded trials showed no obvious differences with the trials in which either eyes or pits were occluded although the strike angles were biased to the open sensory sides. These results are consistent with anatomical and physiological studies indicating that information from both visual and IR sensors are integrated in the ipsilateral tectum. It is also possible that the integration of visual and IR inputs may occur within the brainstem nucleus isthmi of pit vipers insofar as studies in barn owls indicate that this structure is not only a visual processor, but also codes auditory information in a spatiotopic manner [20]. Additional physiological studies are needed to evaluate the role of the nucleus isthmi in pit vipers.

Physiological studies have shown that tectal bimodal neurons may be affected in an inhibitory way by visual and IR input including IR neurons which are depressed by visual input and visual neurons depressed by IR input [15]. Furthermore in many vertebrate taxa connections between the left and right tecta via the tectal commissure include both excitatory and inhibitory components [21], [22], [23]. Another possible explanation for poor performance in the contralateral occlusion condition concerns the possibility that multimodal tectal networks in *Gloydius* perform on the basis of “winner takes all” when inputs from different sensory modalities differ as has been shown in other species [24]. Thus contralateral activation of the visual and IR senses will activate different populations of tectal neurons maximally on each side of the midbrain leading to reduced responding in the contralateral occlusion situation.

An alternative explanation for the poor performance of the snakes in the contralateral occlusion conditions is that the descending tectospinal pathways are also crossed. Thus stimulation of each side of the midbrain with different and non-overlapping sensory input may tend to provoke conflicting response orientation movements. Interference might have occurred if objects (e.g. prey, moving stone or roaming leaf) were located within the overlapping fields of the opened eye and contralaterally open pit. In such cases the sensory images formed on each side of the optic tectum would not be in register because they would involve different sensory systems [13] and might primarily inhibit rather than mutually excite the same bimodal neurons [15]. The adaptive significance of the inhibitory mechanism would lie in the fact that it ensures the snake does not strike when inappropriate visual stimuli (such as a falling leaf) and infrared stimuli (such as stones heated by the sun) occur simultaneously on opposite sides of the head.

In summary the results reported in the present study support the idea that *Gloydius* can hunt efficiently with either the visual or IR sense although performance is significantly better if both senses are available. A novel result showing that interference obtains if visual and IR information are available from sensory organs on opposite sides of the head shows that inhibitory or competing mechanisms may exist. The underlying neural basis of these mechanisms and possible role in natural prey targeting and capture remain to be elucidated in future experiments.

## Materials and Methods

### Animals

**Figure 4:**
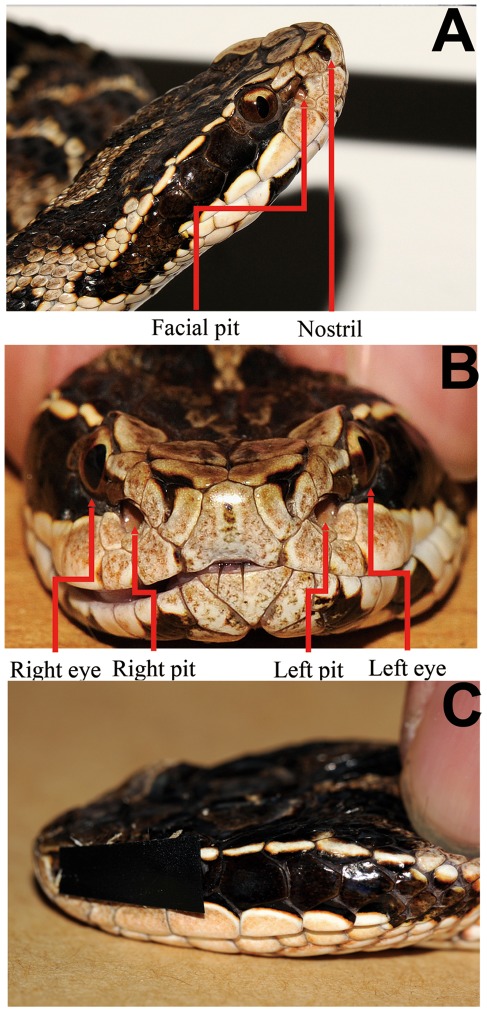
Positions of pits and eyes and demonstrate of sensory occlusion. A: Photograph of the head of *Gloydius brevicaudus* showing the location of the lateral facial pit organ between the ipsilateral eye and nostril. B: Photograph of the rostral view of the head of *G. brevicaudus* showing both eyes and facial pits. C: A photograph of an experimental subject illustrating left side sensory occlusion (see Materials and Methods for explanation).

The short-tailed pit viper, *Gloydius brevicaudus* (Viperidae: Crotalinae; Fig. 4), was selected for study because it is an effective hunter under a variety of conditions including both in light and darkness. *Gloydius* is a relatively small pit viper, easy to control in the experimental arena, remains tranquil in the absence of stimulation, and displays prey orientation and capture behaviors that can be quantified easily under laboratory conditions. Subjects used in this study were collected from Anhui and Hubei provinces in China. All animal work of this paper has been conducted according to relevant national and international guidelines. All animal care and experimental procedures were approved by the Chengdu Institute of Biology Animal Care and Use Committee. No animal suffered unnecessary pain in experiments. Each snake was kept in a home cage, a plastic terrarium (50×35×20 cm), the floor of which was covered with old newspaper (i.e. lacking the smell of printer’s ink), at temperatures between 24–26°C. Water was available *ad libitum*. Prior to the experiment, snakes were fed live mice once every two weeks. For the experiments, 10 subjects with reliable propensities to attack prey were selected (total length of 43–55 cm; weight 45.8–96.6 g; male:female = 5∶5), based on prior observations of their successful predatory behavior. Prey consisted of mice (*Mus musculus*) of both sexes which were used for both normal feeding of the snakes and for the experiments. Suitable mice were chosen according to the body size of each snake (i.e. the width of the head of the mouse was not substantially larger than the size of the head of the snake). The experimental trials were conducted according to the snakes’ normal feeding schedule. In other words, mice used in the targeting trials were part of the normal feeding regimen for the experimental subjects.

### Ocular and Pit Organ Occlusion

Some previous studies focused on the predatory behaviors of snakes with the eyes and/or pits occluded [16], [25]. In this study, we used similar methods to block the visual/IR sensors. To prevent discomfort to the snake while completely covering one or both eyes, skin-friendly medical tape was used as an internal layer and dark electrical tape was used as an opaque external layer. To occlude the pit organs, water-soaked tissue balls (about 1 mm in diameter) were inserted into the pits without injuring the pit membrane, and then covered with medical tape (Fig. 4C). This technique is effective for blocking detection of thermal cues as revealed by extracellular single-unit recording tests (unpublished observations). The tape used for both eye and pit occlusion was trimmed to fit the sensory organ window of each snake.

In order to explore the roles of visual and IR information in prey targeting, the following experimental conditions were employed: (1) control with no sensory occlusion; (2) occlusion of both eyes, (3) occlusion of both pits, (4) occlusion of one eye and one pit on the same side of the head, on either the left (4) or right (5) sides, (6) occlusion of the left eye and right pit, (7) occlusion of the right eye and left pit, (8) occlusion of both eyes with only the left pit open and (9) occlusion of both eyes with only the right pit open, as shown in Table 1. In view of the fact that individual differences might exist and given the limited number of animals, all 10 snakes were used twice for each of the nine experimental conditions, yielding a total of 180 trials.

### Targeting Trials

**Figure 5:**
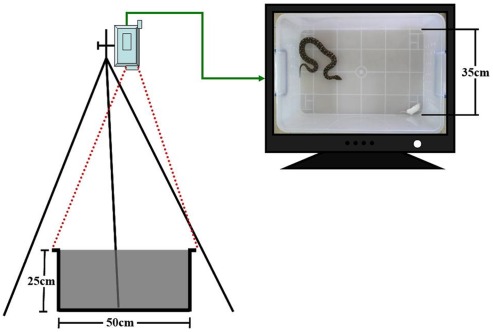
Demonstration of experiment and recording. A schematic diagram illustrating the experimental set-up used to measure behavior during targeting trials. A camcorder mounted on a tripod and connected to a PC was used to record the behavior of the snakes. A grid superimposed on the image was used to quantify strike distances and angles. At the beginning of each trial a mouse of appropriate size was placed directly in front of the head of the snake at the edge of the arena (see Materials and Methods for explanation).

All experimental trials took place in the snakes’ home cages in order to minimize possible effects of changing the environment on the pit vipers’ response to the prey. For the experimental trials, feedings were staged in a soundproof room, videotaped, and monitored by experimenters behind a screen (see Figure 5). During the experiment, the temperature was held constant at 25°C and two energy efficient bulbs (5 W, Philips) were used to light the room. All targeting trials were monitored visually by the experimenters in order to ensure that neither the mice nor snakes suffered unnecessary pain.

For each experimental trial, the choice of snake and the sensory occlusion condition were selected randomly, using a Matlab random number generator. After occlusion of the sensory organs (if necessary), the snake was isolated in its cage for 20 minutes to accommodate to the occlusion. The mouse was then placed directly in the line of sight relative to the center of the snake’s head at the edge of the arena. The experimental set-up is depicted schematically in Figure 5.

A JVC GZ-HD300SAC HD video camera, mounted on a tripod 1 m above the center of the trial arena, was used to record trials (see Figure 5). Data on the hard drive of the video camera were downloaded to a PC (Lenovo, China) for analysis. The following data were recorded or measured for each trial, as detailed for each sensory occlusion condition in Table 1: (1) the number of trials during which there were strikes; (2) the number of times the snake attempted to strike the prey, regardless of whether the strike was successful; (3) the number of successful (effective ) strikes during which the prey was bitten; (4) the strike frequency which equals the mean number of strikes per trial in which at least one strike was launched; (5) the strike latency (s) which equals the time interval from placing the mouse in the arena to launching of the first strike by the snake. Note that for trials with multiple strikes latency was defined as time before the first strike was launched; (6) the strike distance (cm) which is the distance between the center of the prey’s body and the tip of the snake’s rostrum immediately before the strike; and (7) the strike angle (°) which is the head angle immediately before the strike (i.e. the attack), defined as the angle formed between the midline of the snake’s head and the center of the body of the prey. For strike angle, deviation to the right was scored as positive and deviation to the left was scored as negative. The last two parameters were the same as those used in previous studies [25], [26]. In order to facilitate comparison of the results presented here with these previous studies the same type of polar coordinate diagrams were used to represent the data (see Figs 1, 2). Finally, data from all conditions were scored by an investigator blind to the experimental condition.

During testing, if the snake did not strike the mouse by the end of the 20-minute trial, the occlusion materials were removed and the snake was provided with an opportunity to feed in accordance with the normal feeding regimen.

### Statistical Analyses

Prior to the statistical analyses, all data were examined for assumptions of normality and homogeneity of variance, using the Kolmogorov-Smirnov and Levene tests, respectively. Two parameters, the number of strikes and latency time, failed to pass these tests and were analyzed with non-parametric tests. In order to avoid the effects of snake bias on the results, Pearson and Spearman correlation coefficients were used to test whether there were correlations between snakes and parameters. No such correlations were found between individuals (including snakes and mice) and the parameters tested (*P*>>0.05 in each case). Wilcoxon signed-ranks tests were used to evaluate differences between conditions and to determine whether average strike angles differed from 0° within each condition for the variables assessed in this study. Tabled values are expressed as mean ± s.d.; *P*<0.05 was considered significant and *P*<0.005 was considered extremely significant in each case. All statistical tests were performed using SPSS (version 13.0 for Windows) and the polar coordinate plots of individual strikes were created using SigmaPlot (version 11.0 for Windows).
